# Identification of potential diagnostic biomarkers for tenosynovial giant cell tumour by integrating microarray and single-cell RNA sequencing data

**DOI:** 10.1186/s13018-023-04279-2

**Published:** 2023-11-29

**Authors:** Chen Chen, Linli Zheng, Gang Zeng, Yanbo Chen, Wenzhou Liu, Weidong Song

**Affiliations:** 1 Department of Orthopedics, Sun Yat-Sen Memorial Hospital, Sun Yat-Sen University, Yingfeng Road, 33rd, Haizhu District, Guangzhou, 510000 Guangdong Province China; 2 Joint Surgery, The First Affiliated Hospital, Sun Yat-Sen University, No.58 Zhongshan Er Road, Guangzhou, 510080 Guangdong Province China

**Keywords:** Tenosynovial giant cell tumour, Osteoclast, Bioinformatics analysis, Gene Expression Omnibus

## Abstract

**Purpose:**

Tenosynovial giant cell tumour (TGCT) is a benign hyperplastic and inflammatory disease of the joint synovium or tendon sheaths, which may be misdiagnosed due to its atypical symptoms and imaging features. We aimed to identify biomarkers with high sensitivity and specificity to aid in diagnosing TGCT.

**Methods:**

Two scRNA-seq datasets (GSE210750 and GSE152805) and two microarray datasets (GSE3698 and GSE175626) were downloaded from the Gene Expression Omnibus (GEO) database. By integrating the scRNA-seq datasets, we discovered that the osteoclasts are abundant in TGCT in contrast to the control. The single-sample gene set enrichment analysis (ssGSEA) further validated this discovery. Differentially expressed genes (DEGs) of the GSE3698 dataset were screened and the Gene Ontology (GO) and Kyoto Encyclopedia of Genes and Genomes (KEGG) pathway enrichment analyses of DEGs were conducted. Osteoclast-specific up-regulated genes (OCSURGs) were identified by intersecting the osteoclast marker genes in the scRNA-seq and the up-regulated DEGs in the microarray and by the least absolute shrinkage and selection operator (LASSO) regression algorithm. The expression levels of OCSURGs were validated by an external dataset GSE175626. Then, single gene GSEA, protein–protein interaction (PPI) network, and gene-drug network of OCSURGs were performed.

**Result:**

22 seurat clusters were acquired and annotated into 10 cell types based on the scRNA-seq data. TGCT had a larger population of osteoclasts compared to the control. A total of 159 osteoclast marker genes and 104 DEGs (including 61 up-regulated genes and 43 down-regulated genes) were screened from the scRNA-seq analysis and the microarray analysis. Three OCSURGs (MMP9, SPP1, and TYROBP) were finally identified. The AUC of the ROC curve in the training and testing datasets suggested a favourable diagnostic capability. The PPI network results illustrated the protein–protein interaction of each OCSURG. Drugs that potentially target the OCSURGs were predicted by the DGIdb database.

**Conclusion:**

MMP9, SPP1, and TYROBP were identified as osteoclast-specific up-regulated genes of the tenosynovial giant cell tumour via bioinformatic analysis, which had a reasonable diagnostic efficiency and served as potential drug targets.

**Supplementary Information:**

The online version contains supplementary material available at 10.1186/s13018-023-04279-2.

## Introduction

Tenosynovial giant cell tumour (TGCT), of which there is a localized and a diffuse type, is a monoarticular, mesenchymal lesion of the joint synovium or tendon sheaths [1, 2], characterized by both neoplastic and inflammatory features [3]. Although the diffuse type was called pigmented villonodular synovitis (PVNS) in the past, TGCT has been proposed to replace both designations in the latest version of the World Health Organization classification [4]. The symptoms of TGCT, such as pain, tenderness, swelling, or limitation of motion, are unspecific, causing magnetic resonance imaging (MRI) and pathological biopsy necessary to make a proper diagnosis [5]. The localized TGCT appears as a focal mass that usually abuts or surrounds the tendon while joint effusion or synovial fluid is typical in the diffuse type [6, 7]. Sometimes TGCT mimics other soft tissue tumours on MRI, making diagnosis challenging [8, 9].

Microarray analysis is a powerful tool to uncover gene expression differences between disease conditions and controls whereas single-cell RNA sequencing (scRNA-seq) helps reveal transcriptome heterogeneity between cells. Several studies have combined them to construct a prognosis model [10] and show the landscape of the immune microenvironment [11] and the mechanisms of biological processes [12]. Using sequencing techniques, researchers revealed that osteoclastogenesis and osteoclast differentiation are vital characteristics of TGCT [13, 14]. However, no studies explore the possibility of diagnosing TGCT using osteoclast-specific genes.

In this study, we integrated two scRNA-seq datasets [14, 15] and two microarray datasets [13, 16] from Gene Expression Omnibus (GEO) dataset to identify osteoclast-specific up-regulated genes (OCSURGs) to predict the diagnosis of TGCT. Up-regulated genes were screened from the discovering cohort (GSE3698, GSE210750, and GSE152805). Three OCSURGs (MMP9, SPP1, TYROBP) were identified after making an intersection and the least absolute shrinkage and selection operator (LASSO) regression. Receiver operating characteristic (ROC) curves and area under the curve (AUC) of these OCSURGs suggest a good diagnostic value. The protein–protein interaction (PPI) network and single-gene gene set enrichment analysis (GSEA) were used to probe their possible protein interaction and pathway. Finally, the gene-drug network was built to explore the potential drugs targeting three OCSURGs.

## Materials and methods

### Datasets selection

Two microarray datasets (GSE3698 and GSE175626) and two scRNA-seq datasets (GSE210750 and GSE152805) were selected. GSE3698 includes synovial membrane tissues from osteoarthritis (OA) patients (n = 19), rheumatoid arthritis (RA) patients (n = 18), and TGCT patients (n = 11). GSE175626 contains three synovial membrane tissues from OA patients and three synovial membrane tissues from TGCT patients. GSE210750 includes three TGCT lesions. GSE152805 includes synovial membrane tissues from OA patients (n = 3). Data from the RA synovial membrane was removed for further analysis. OA synovial membrane was considered as the control in this study.

### Microarray analysis

The gene expression matrix of microarray datasets was downloaded from the GEO. Probes were transformed into gene symbols according to the annotation profile of each dataset. The average expression of the duplicated genes was calculated for further analysis. Differentially expressed genes (DEGs) were screened using the “limma” R package [17]. Genes with |log2FC|> 0.5 and adjusted *P*-value (adj. P. Val.) < 0.05 were considered DEGs.

### GO and KEGG pathway enrichment analyses

The gene names of DEGs were converted to Entrez ID. Gene Ontology (GO) and Kyoto Encyclopedia of Genes and Genomes (KEGG) pathway enrichment analysis of the DEGs were performed using the “clusterProfiler” R package [18]. The enriched pathway with a *p*-value < 0.05 was considered significant.

### Quality control of scRNA-seq datasets

The Seurat package (version 4.3.0) [19] was utilized for quality control and further analysis. The barcodes, features, and matrix files of each scRNA library were read into R using the Read10X function to create Seurat objects. All Seurat objects were merged into an integrated one after renaming the cell label with the RenameCells function. Cells with less than 200 and more than 6000 detected features, and those with > 20% mitochondrial genes were deleted (Additional file 1: Fig. S1A).

### Analysis of scRNA-seq

The “FindVariableFeatures” method was applied to extract genes with high intercellular variability, and the top 1200 genes with significant fluctuations were extracted for subsequent analysis. The batch effect was removed using the Harmony package [20] (Additional file 1: Fig. S1C, D). Doublets predicted by the DoubletFinders R package [21] were filtered (Additional file 1: Fig. S1E). Finally, cell clusters were identified by running the RunUMAP function, the FindNeighbors function, and the FindClusters function, with a dimension setting of 1:20 and a resolution setting of 0.6.

### ssGSEA analysis

The ssGSEA algorithm is an extension of the Gene Set Enrichment Analysis (GSEA) that calculates separate enrichment scores for each pairing of a sample and gene set. Osteoclast marker genes detected by the scRNA-seq analysis were integrated into the feature gene panels from a previous study [22]. The gene expression matrix of the GSE3698 dataset and the integrated gene panels were used as input files. Then the infiltrating scores of 28 immune cells as well as osteoclasts in the were calculated by running the “gsva” function in the "GSVA" R package [23] with the “method” parameter set to “ssGSEA” and other parameters set to default. This computational approach allows researchers to characterize tumour-infiltrating cells in the microarray or bulk RNA sequencing dataset.

### ROC curve

The receiver operating characteristic (ROC) analysis was performed to examine the sensitivity and specificity of three OCSURGs using the “pROC” R package [24]. The area under the curve (AUC) was calculated. The AUC ranges from 0.5 to 1. The closer the AUC is to 1, the higher the predictive ability.

### Statistical analysis

All statistical analysis was performed using R software (version 4.2.1). Wilcoxon test was conducted to compare the difference between groups. *P* value < 0.05 was considered statistically significant.

## Result

### Cells clustering and annotation of scRNA-seq data

**Fig. 1:**
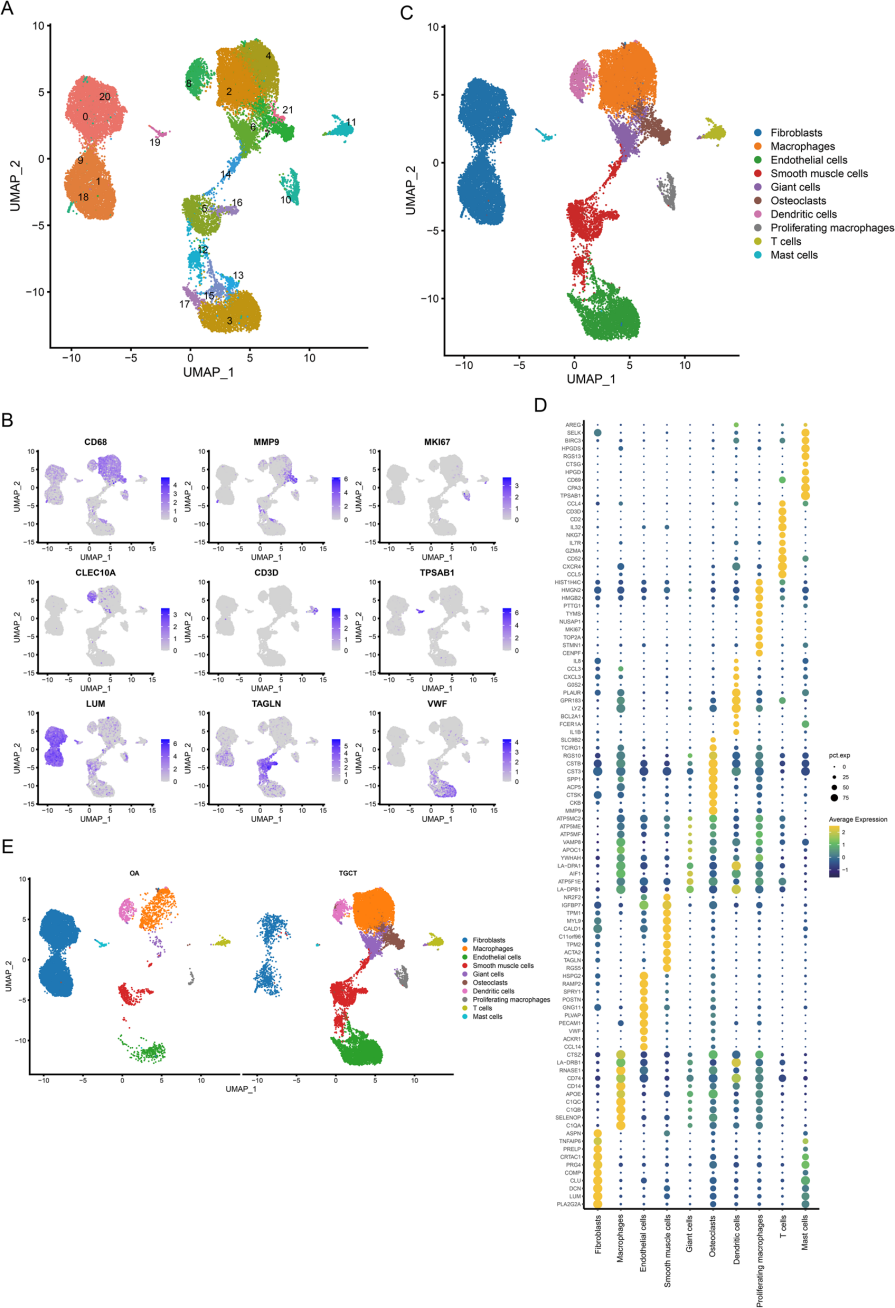
Single-cell profiling of TGCT lesions and OA synovial membrane. **A** UMAP projection of 27314 cells which were clustered into 19 clusters. **B** Feature plots showing the expression level of canonical genes. **C** UMAP projection of 10 cell types. **D** Dotplot showing the top 10 marker genes of each cell type. **E** UMAP projection of all cells presenting in different groups

**Fig. 2:**
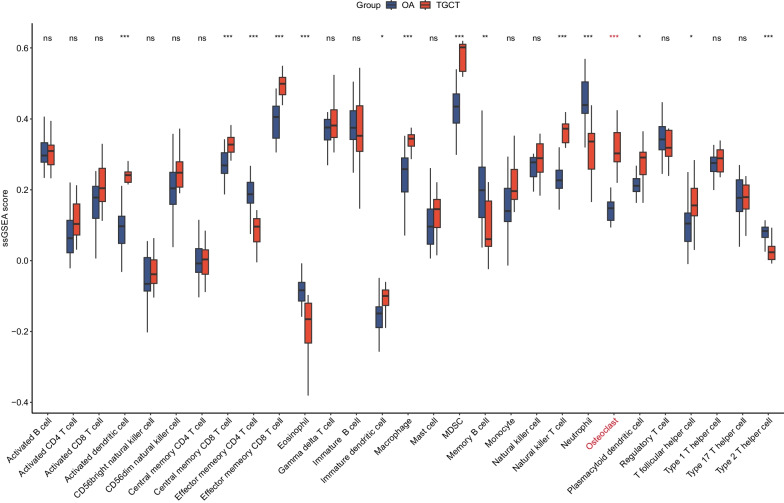
ssGSEA analysis of the GSE3698 dataset. *p-value < 0.05; **p-value < 0.01; ***p-value < 0.001; ns, not significant

Two scRNA-seq datasets of 10 × Genomics were integrated to explore the cellular component of TGCT and OA. The GSE210750 dataset contains 3 TGCT lesions which generated 5 libraries, and the GSE152805 contains 3 synovium samples from OA. A high correlation coefficient between the cell counts and genes was calculated (R = 0.91) but not observed between the cell counts and mitochondrial genes (R =  − 0.48) (Additional file 1: Fig. S1B). A total of 27,314 cells were included after quality control and clustered into 22 Seurat clusters (Fig. 1A). 9 cell types were manually annotated according to the expression level of canonical genes (Fig. 1B, C and Table 1). Then we performed Spearman correlation analysis to compare the transcriptome similarities between each Seurat cluster (Additional file 1: Fig. S1F). The correlation coefficient between Seurat cluster 6 and Seurat cluster 7 (annotated as osteoclasts) is 0.97. Given that, we annotated Seurat cluster 6 as giant cells, which expressed osteoclast phenotype in TGCT [25]. Afterwards, we run the FindAllMarkers function with a logfc setting of 0.5 to screen marker genes of each cell type. The top 10 marker genes of each cell type are shown in Fig. 1D. It was worth mentioning that the TGCT samples show a greater abundance of osteoclasts and giant cells (Fig. 1E), suggesting their potential roles in TGCT. What’s more, we deconvoluted the GSE3698 dataset by ssGSEA showing that the score of osteoclast, activated dendritic cell, immature dendritic cell, central memory CD8 T cell, effector memory CD8 T cell, macrophage, myeloid-derived suppressor cell (MDSC), natural killer T cell, plasmacytoid dendritic cell, and T follicular helper cell was significantly higher in TGCT than in OA. The score of effector memory CD4 T cell, type 2 T helper cell, memory B cell, eosinophil, and neutrophil was significantly lower in TGCT than in OA (Fig. 2). The score derived from ssGSEA reflects the degree to which the input gene set is coordinately up- or down-regulated within a sample, thus representing the density of infiltrating cells. We further validated the ssGSEA score in the GSE175626 dataset and it also showed that the ssGSEA score of osteoclasts was higher in TGCT than in OA (Additional file 2: Fig. S2). However, there was no statistical difference due to the insufficiency of sample size. In a word, we discovered that the infiltration of osteoclasts was a key feature of TGCT.

**Table 1:** Annotation of Seurat clusters

Seurat clusters	Cell types	Canonical marker genes
2, 4	Macrophages	CD68
10	Proliferating Macrophages	CD68, MKI67
7, 21	Osteoclasts	MMP9
8	Dendritic cells	CLEC10A
11	T cells	CD3D
19	Mast cells	TPSAB1
0, 1, 9, 18, 20	Fibroblasts	LUM
5, 12, 14, 16	Smooth muscle cells	TAGLN
3, 13, 15, 17	Endothelial cells	VWF

### Identification of differentially expressed genes (DEGs) in the microarray

**Fig. 3:**
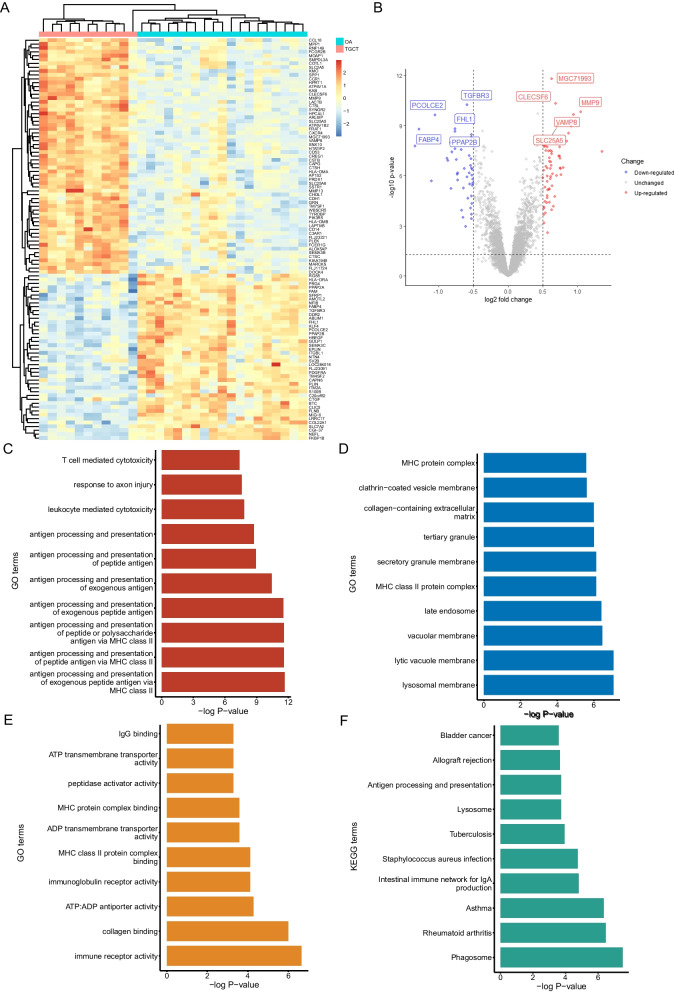
Identification and enrichment analysis of the DEGs. Heatmap (**A**) and volcano plot (**B**) of 104 DEGs between TGCT lesion and OA synovial membrane. Top 10 GO terms including BP (**C**), CC (**D**) and MF (**E**) of DEGs. (**F**) Top 10 KEGG terms of DEGs

The gene expression matrix of the GSE3698 dataset was downloaded from the GEO and read into R software. After transforming the probe ID and calculating the mean expression of duplicated genes, we used the ‘limma’ package to identify a total of 104 DEGs, including 61 up-regulated genes and 43 down-regulated genes (Fig. 3A and B).

### Functional enrichment analysis of DEGs

GO enrichment analysis and KEGG enrichment analysis were performed to explore the potential function of the DEGs. The GO terms include biological processes (BP), cellular components (CC), and molecular functions (MF). For BP, the DEGs were mainly enriched in antigen processing and presentation, leukocyte mediated cytotoxicity, response to axon injury, and T cell mediated cytotoxicity (Fig. 3B). For CC, the DEGs were mainly enriched in lysosomal membrane, lytic vacuole membrane, and vacuolar membrane (Fig. 3D). The top three terms of MF were immune receptor activity, collagen binding, and ATP: ADP antiporter activity (Fig. 3E). The KEGG enrichment analysis shows that the DEGs were related to phagosome, rheumatoid arthritis, and asthma (Fig. 3F).

### Identification of OCSURGs in TGCT

**Fig. 4:**
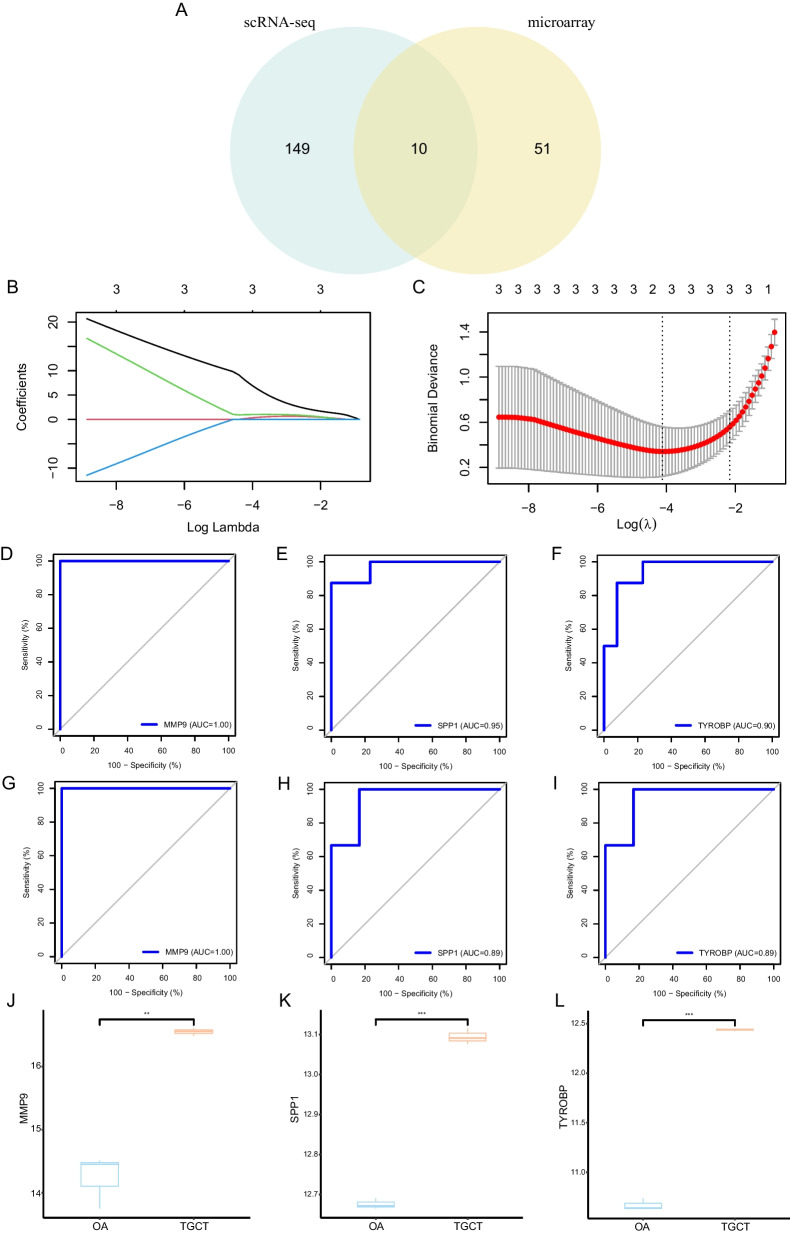
Identification and validation of OCSURGs. **A** Venn plot showing the intersection of up-regulated DEGs from microarray analysis and osteoclast marker genes from scRNA-seq. **B**, **C** LASSO algorithm retains three OCSURGs from 10 intersected genes. **D**, **E**, **F** The ROC curves of MMP9, SPP1, and TYROBP in the training dataset. **G**, **H**, **I** The ROC curves of MMP9, SPP1, and TYROBP in the testing dataset. **J**, **K**, **L** The expression level of MMP9, SPP1, and TYROBP in the GSE175626 dataset. *p-value < 0.05; **p-value < 0.01; ***p-value < 0.001; ns, not significant

We made an intersection of the up-regulated DEGs from the microarray analysis and osteoclast marker genes from the scRNA-seq analysis to yield osteoclast-specific up-regulated genes (OCSURGs) in TGCT. A total of ten overlapped genes (MMP9, ATP6V1B2, ATP6V1A, SPP1, LAPTM5, TYROBP, CSTB, SNX10, CCR1, and GRN) were identified (Fig. 4A). Then we divided the GSE3698 dataset into training and testing cohorts in a 7:3 ratio. The least absolute shrinkage and selection operator (LASSO) regression algorithm was utilized to narrow down these genes (Fig. 4B and C). After tenfold cross-validation, the lambda that gives minimal mean cross-validated error was 0.016. Three OCSURGs (MMP9, SPP1, TYROBP) were retained by increasing the penalty parameter (λ). The diagnostic efficacy of the OCSURGs was validated in the training dataset and testing dataset. The AUC of the ROC curve in the training dataset was 1.00, 0.95, and 0.90, separately (Fig. 4D–F), while the value in the testing dataset was 1.00, 0.89, and 0.89(Fig. 4G–I), suggesting a favourable diagnostic capability. What’s more, the OCSURGs were also significantly up-regulated in the GSE175626 dataset (Fig. 4J–L). However, since the GSE175626 dataset only consists of three TGCT lesions and three OA synovial membrane tissues, an external dataset with a larger sample is needed.

### Enrichment analysis, PPI network, and gene-drug network of OCSURGs

**Fig. 5:**
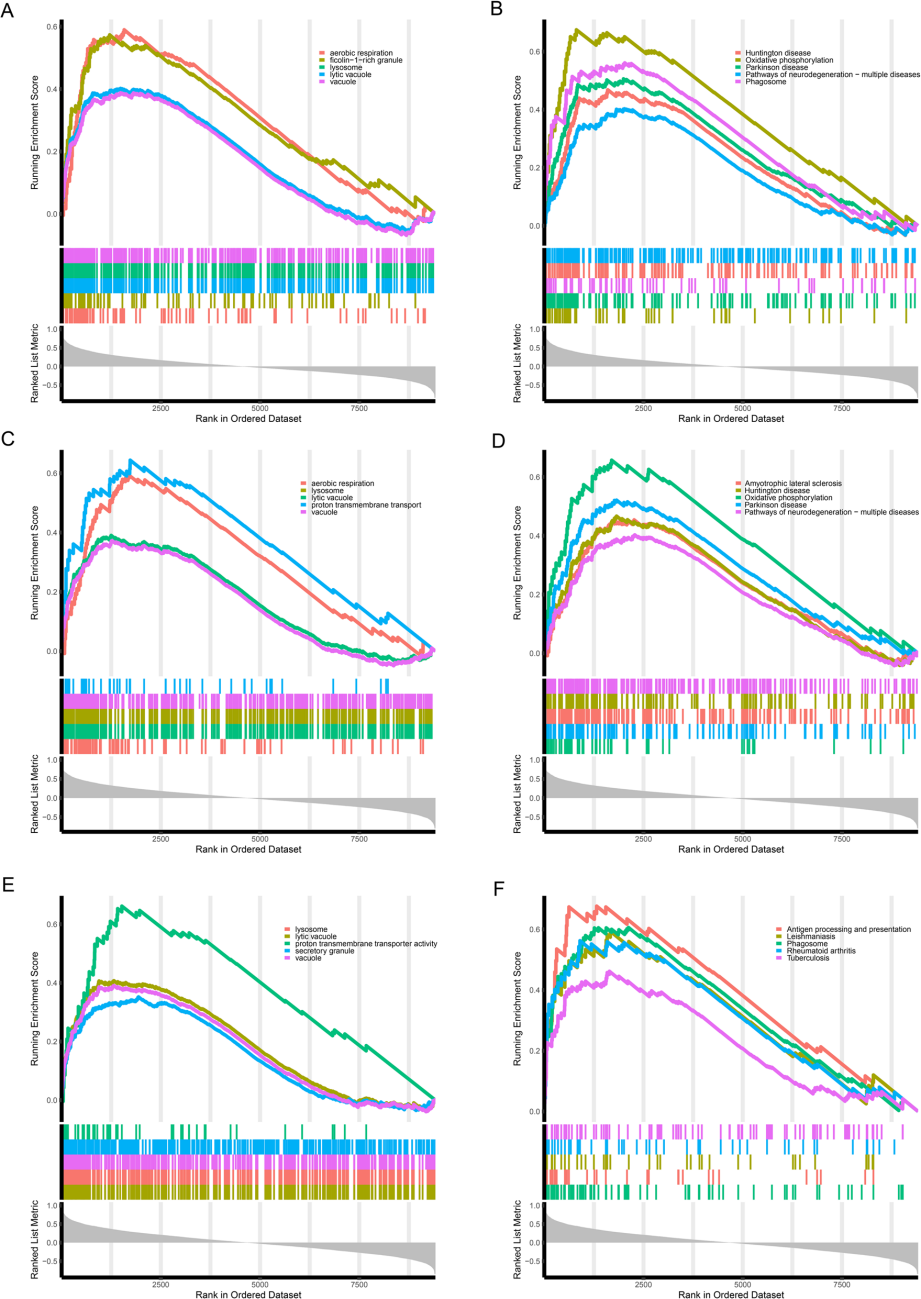
The **A** GO and **B** KEGG terms from single-gene GSEA for MMP9. The **C** GO and **D** KEGG terms from single-gene GSEA for SPP1. The **E** GO and **F** KEGG terms from single-gene GSEA for TYROBP

**Fig. 6:**
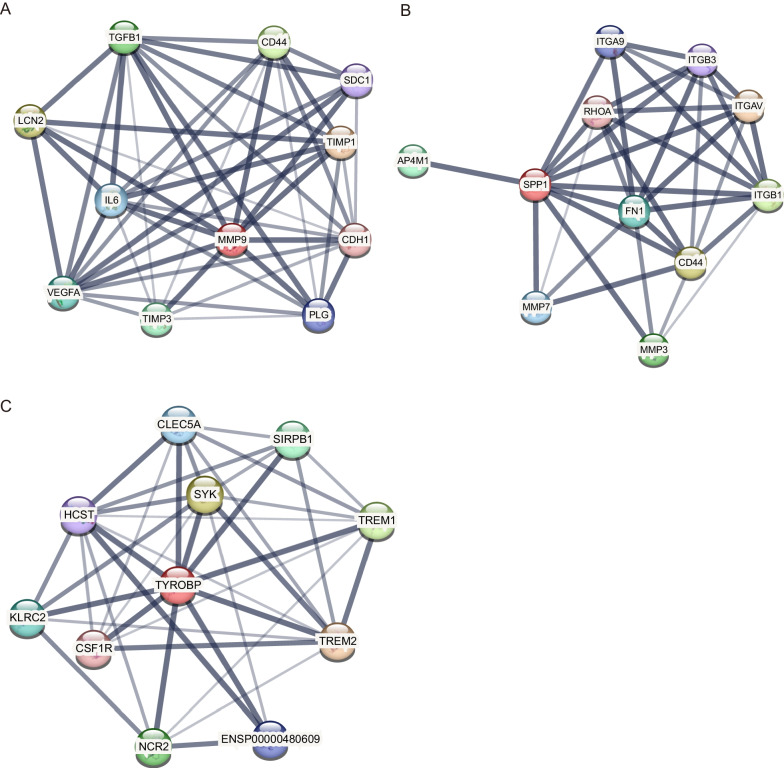
PPI network of **A** MMP9, **B** SPP1, and **C** TYROBP

To understand the latent pathway of each OCSURG, we performed the single gene GSEA analysis. The top five pathways of GO and KEGG for three OCSURGs are shown in Fig. 5. Then we imported the OCSURGs to the STRING database to construct protein–protein interaction (PPI) network (Fig. 6). The edges indicate functional as well as physical protein connection, while the line thickness implies the strength of data support. Through the DGIdb database, we investigated drugs targeting the OCSURGs (Table 2). Carboxylated Glucosamine, Andecaliximab, Marimastat, Curcumin Pyrazole, Incyclinide, S-3304, Tozuleristide, Demethylwedelolactone, Prinomastat, Bevacizumab, Celecoxib, and Curcumin targeted MMP9. ASK-8007, Calcitonin, Alteplase, Gentamicin, Wortmannin, and Tacrolimus targeted SPP1. However, no predicted drug was obtained for TYROBP from the DGIdb database.

**Table 2:** The predicted drugs targeting the two OCSURGs

Gene	Drug	Interaction types & directionality	Sources	Reference (PMID)
MMP9	Carboxylated Glucosamine	n/a	DTC	16,616,490
MMP9	Andecaliximab	antibody (inhibitory), inhibitor (inhibitory)	ChemblInteractions/TTD	None found
MMP9	Marimastat	inhibitor (inhibitory)	TdgClinicalTrial/ TEND	12,763,661, 17,234,180, 11,752,352
MMP9	Curcumin Pyrazole	n/a	DTC	19,128,977
MMP9	Incyclinide	n/a	TdgClinicalTrial	None found
MMP9	S-3304	vaccine (activating)	TALC	None found
MMP9	Tozuleristide	n/a	TTD	None found
MMP9	Demethylwedelolactone	n/a	DTC	22,926,226
MMP9	Prinomastat	vaccine (activating)	TALC	None found
MMP9	Bevacizumab	n/a	CIVic	26,921,265
MMP9	Celecoxib	n/a	PharmGKB	22,336,956
MMP9	Curcumin	n/a	TTD	None found
SPP1	ASK-8007	inhibitor (inhibitory)	ChemblInteractions	None found
SPP1	Calcitonin	n/a	NCI	8,013,390
SPP1	Alteplase	n/a	NCI	12,009,309
SPP1	Gentamicin	n/a	NCI	11,274,264
SPP1	Wortmannin	n/a	NCI	14,703,434
SPP1	Tacrolimus	n/a	NCI	16,103,732

## Discussion

Tenosynovial giant cell tumour (TGCT) is a common benign soft tissue tumour characterized by immune cell infiltration, synoviocytes hyperproliferation, and accumulation of monocyte-derived osteoclasts in the synovial tissue of the joint [25]. Monocytes and stromal cells produce cytokines such as tumour necrosis factor α (TNF-α), interleukin (IL)-6, and IL-1 to activate osteoclast, causing bone destruction and matrix degradation [26, 27]. The accumulated giant cells in TGCT also show osteoclastic features [27–29]. Due to its atypical symptoms, the diagnosis of TGCT is sometimes challenging. MRI is a preferred imaging tool for diagnosing soft tissue tumours because of its multiplanarity and optimal tissue contrast resolution [30]. However, it may be hard to distinguish TGCT from other soft tissue lesions if there is a low presence of blooming artefact, which is a characterization of TGCT on gradient echo (GRE) sequences [31], and misdiagnoses may occur [8, 9]. Kim et al. [32] constructed an MRI prediction model for diffuse TGCT based on a relatively small sample size and retrospective design. Zhao et al. [33] reported fine needle aspiration cytology (FNAC) may be useful in distinguishing TGCT from other cytologically similar lesions. Yet no study explores the possibility of using gene signatures to assist in diagnosing TGCT so far.

In our scRNA-sea analysis, we found that osteoclasts were more abundant in TGCT in contrast to OA synovial tissues. Due to the small sample size of the scRNA-seq, we applied ssGESA, a deconvolute method, to validate this observation. We first identified osteoclast marker genes by running the FindAllMarkers function in the Seurat package. A total of 159 osteoclast marker genes were obtained finally. Then these genes were integrated into the feature gene panels from a previous study [22]. The ssGSEA score of each cell type was calculated to represent the relative abundance. Results showed that the proportion of osteoclasts was significantly higher in TGCT than in OA. Then we obtained up-regulated genes in TGCT from microarray analysis and osteoclast marker genes from scRNA-seq analysis. By making the intersection of the above genes, we identified ten overlapped genes (MMP9, ATP6V1B2, ATP6V1A, SPP1, LAPTM5, TYROBP, CSTB, SNX10, CCR1, and GRN). The LASSO regression is a machine learning algorithm that penalizes the variables to prevent overfitting. Three genes (MMP9, SPP1, TYROBP) were eventually retained after lasso regression and defined as osteoclast-specific up-regulated genes (OCSURGs). The single gene GSEA analysis showed that both OCSURGs were related to the lysosome, which is an organelle highly related to bone resorption [34–36].

As an important member of the matrix metalloproteinase family, MMP9 plays an essential role in normal physiological processes, such as embryonic development [37], nervous system development [38], and angiogenesis [39], as well as in disease processes. MMP9 is essential for migrating and recruiting macrophages into the glomerulus in glomerulonephritis [40]. In addition, it has been reported that MMP9 levels were associated with systolic hypertension and arterial stiffness [41]. Our results showed that the AUC value of MMP9 was 1, suggesting a favourable ability to distinguish TGCT from control samples. However, studies with greater sample sizes are needed to confirm this finding. In a recent study [42], researchers inhibited the secretion of MMP9, thus suppressing bone resorption by targeting ATP5B, providing new insight into protecting bones in rheumatoid arthritis (RA). Since osteoclastogenesis is one of the features of TGCT, targeting MMP9 might be a potential therapy. S-3304 is a potent inhibitor of MMP2 and MMP9. A phase 1 clinical trial which enrolled 32 patients with solid tumours showed that S-3304 was extremely well tolerated and produced inhibition of gelatinase activity at a dose that produced little toxicity [43]. Andecaliximab, a monoclonal antibody targeting MMP9, also showed promising clinical activity in phase 1 and phase 2 clinical trials [44, 45].

Secreted phosphoprotein 1 (SPP1), also known as osteopontin, is an extracellular matrix protein involved in many biological processes. It is produced by several cell types, such as immune cells, smooth muscle cells, hepatocytes, neural cells, and cells involved in bone morphogenesis, such as osteoblasts and osteoclasts [46]. A recent study indicated that osteopontin derived from macrophages in epididymal adipose tissue promoted bone resorption [47]. The expression of osteopontin in bone tissue is closely related to the formation and arrangement of collagen fibres. Researchers found that type I collagen fibres in bone were irregularly arranged, and bone mass was significantly reduced by inhibiting the expression of osteopontin, suggesting its important function in bone tissue [48]. Except for its important role in bone morphogenesis, osteopontin was also related to tumour cell proliferation, angiogenesis, and metastasis [49]. Calcitonin is a hormone that is primarily produced by the parafollicular cells of the thyroid gland. Although researchers found that eel calcitonin inhibited osteopontin mRNA expression as well as bone-resorbing activity of isolated rabbit osteoclasts [50], further studies are needed to explore the potential of calcitonin in treating bone erosive disease.

TYROBP gene encodes a transmembrane signalling polypeptide that contains an immunoreceptor tyrosine-based activation motif (ITAM) in its cytoplasmic domain. Together with its receptor, triggering receptor expressed on myeloid cells 2 (TREM2), TYROBP contributes to the onset and progression of Alzheimer's disease by impacting various cellular processes such as phagocytosis, cytokine production, and inflammation [51]. Furthermore, TYROBP regulates both the formation and function of osteoclasts [52, 53]. Inadequate TYROBP/TREM2 signalling leads to a suboptimal and delayed differentiation of osteoclasts, which exhibit a significantly diminished capacity for bone resorption in vitro [54]. Our study showed that the expression level of TREM2 in TGCT is higher than in OA (Additional file 3: Fig. S3A). Besides, it was observed that the expression of TREM2 showed a positive correlation with that of TYROBP in TGCT (Additional file 3: Fig. S3B), but not in the case of OA (Additional file 3: Fig. S3C). These results suggest an underlying role of the TYROBP/TREM2 signalling pathway in regulating osteoclastogenesis in TGCT.

## Conclusion

In summary, we unveiled the difference in the cellular composition of TGCT lesion and OA synovial membrane, explored the potential function of DEGs, and identified three OCSURGs (MMP9, SPP1, and TYROBP) by integrating microarray and scRNA-seq for the first time. Furthermore, these OCSURGs showed reasonable diagnostic efficiency. Our study may contribute to the diagnosis of TGCT and offer insights into the prevention of bone destruction.

### Supplementary Information

**Additional file 1**. **Fig. S1**. Quality control of scRNA-seq. (A) Violin plots showing the quality control of single-cell data. (B) A high correlation coefficient of 0.91 between cell counts and genes was observed, but not in cell counts and mitochondrial genes. (C) UMAP projection of all cells before removing batch effect. (D) UMAP projection of all cells after removing batch effect. (E) Identification of doublets by the DoubletFinders R package. (F) Spearman correlation analysis of Seurat clusters.

**Additional file 2**. **Fig. S2**. ssGSEA analysis of the GSE175626 dataset. *p-value < 0.05; **p-value < 0.01; ***p-value < 0.001; ns, not significant.

**Additional file 3**.** Fig. S3**. (A) The expression level of TREM2 in the GSE3698 dataset. The correlation analysis of the expression of TREM2 and TYROBP in TGCT (B) and OA (C).

## Data Availability

All data used in the present study were available from the GEO, STRING, and DGIdb databases.

## Abbreviations

TGCT: Tenosynovial giant cell tumour
GEO: Gene expression omnibus
scRNA-seq: Single-cell RNA sequencing
ssGSEA: Single-sample gene set enrichment analysis
DEGs: Differentially expressed genes
GO: Gene ontology
KEGG: Kyoto encyclopedia of genes and genomes
OCSURGs: Osteoclast-specific up-regulated genes
BP: Biological processes
CC: Cellular components
MF: Molecular functions
LASSO: Least absolute shrinkage and selection operator
PPI: Protein–protein interaction
ROC: Receiver operating characteristic
AUC: Area under the curve
STRING: Search tool for recurring instances of neighbouring genes
DGIdb: Drugs gene interaction database
