# Supplementary figures and images for: Identification of potential diagnostic biomarkers for tenosynovial giant cell tumour by integrating microarray and single-cell RNA sequencing data
Source: J Orthop Surg Res. 2023 Nov 29;18:905. doi: 10.1186/s13018-023-04279-2 (PMC10685511; doi:10.1186/s13018-023-04279-2)

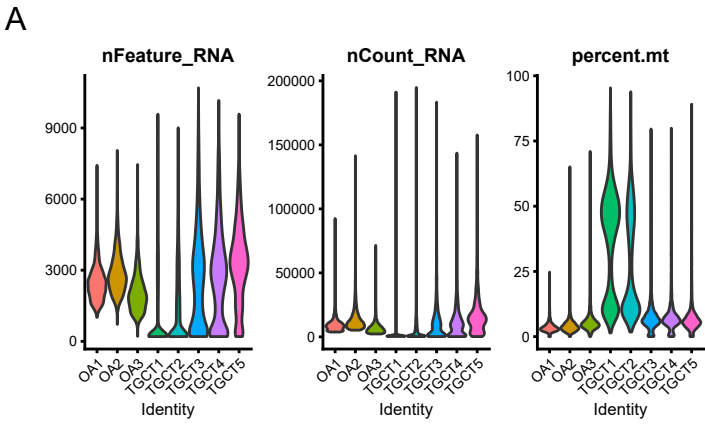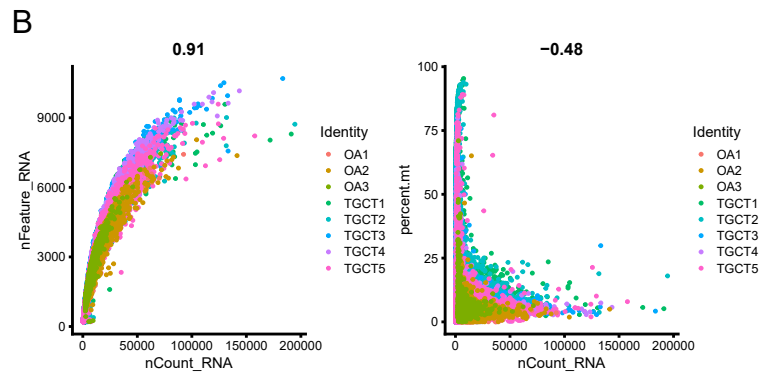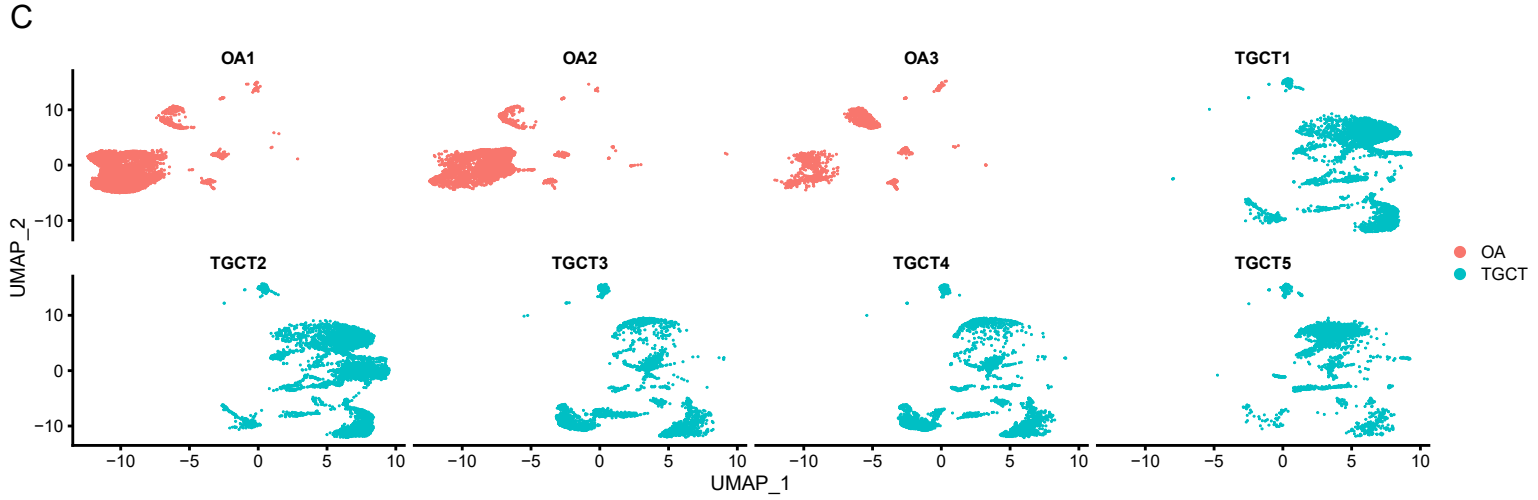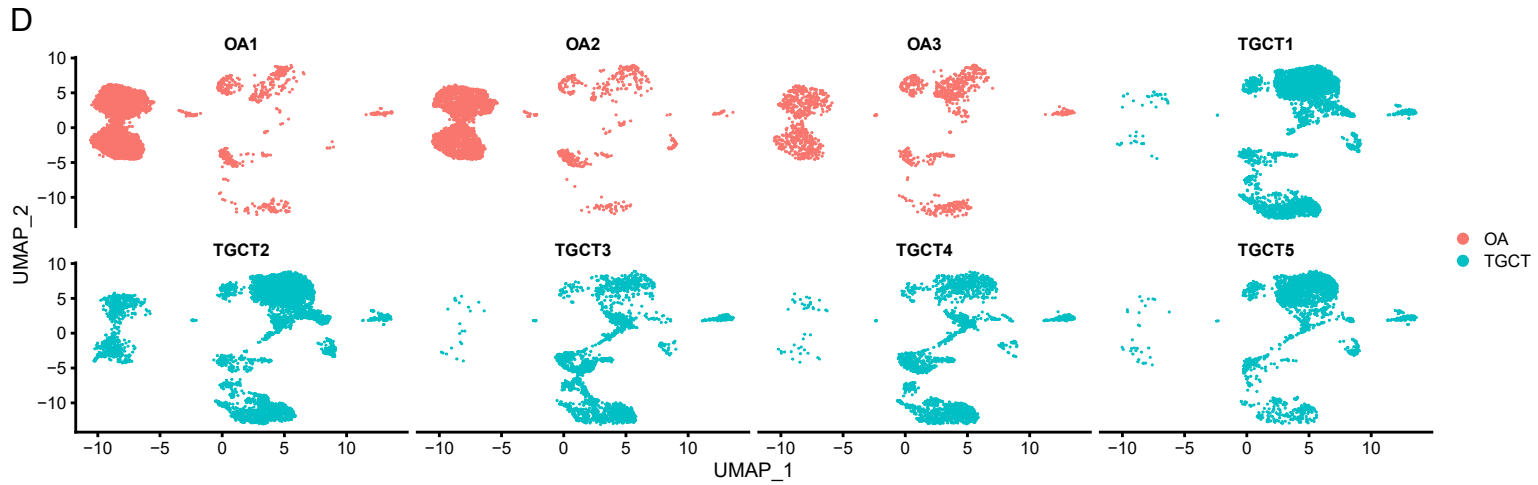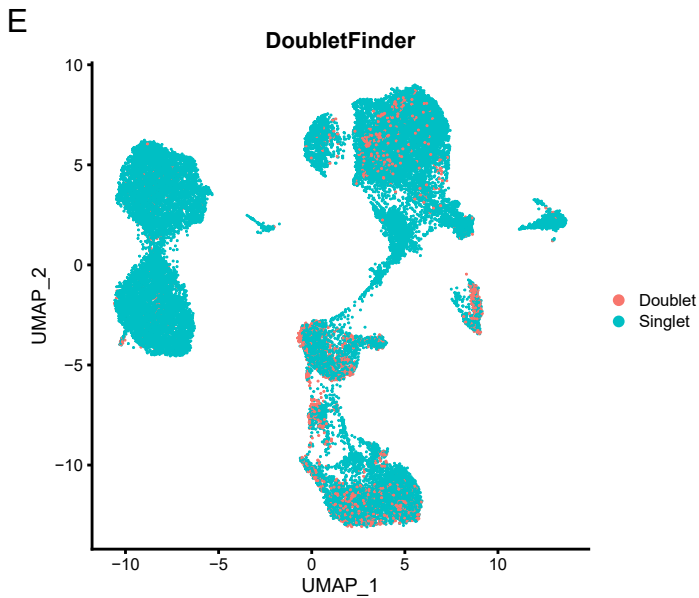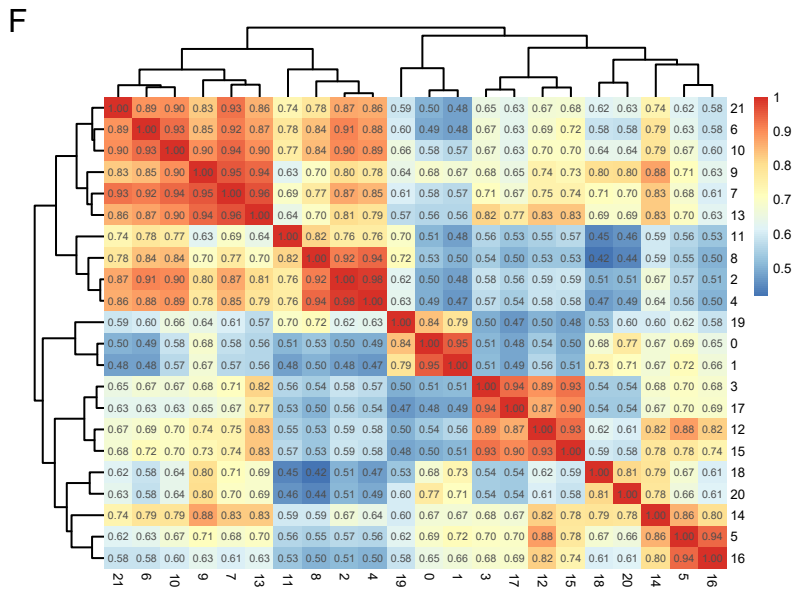

Supplement: Supplementary file 1 — Additional file 1. Fig. S1. Quality control of scRNA-seq. (A) Violin plots showing the quality control of single-cell data. (B) A high correlation coefficient of 0.91 between cell counts and genes was observed, but not in cell counts and mitochondrial genes. (C) UMAP projection of all cells before removing batch effect. (D) UMAP projection of all cells after removing batch effect. (E) Identification of doublets by the DoubletFinders R package. (F) Spearman correlation analysis of Seurat clusters. [file 13018_2023_4279_MOESM1_ESM.pdf]

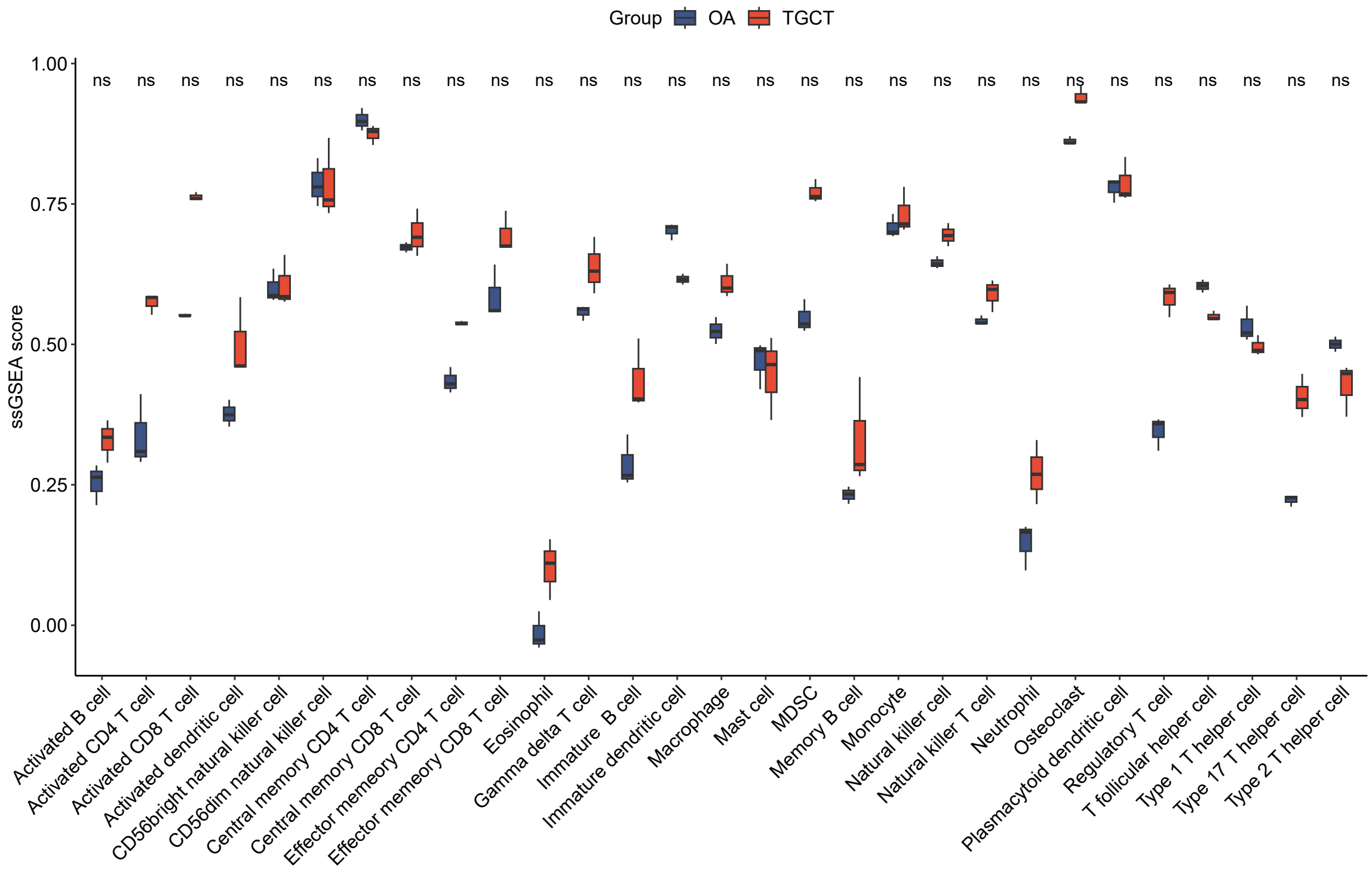

Supplement: Supplementary file 2 — Additional file 2. Fig. S2. ssGSEA analysis of the GSE175626 dataset. *p-value < 0.05; **p-value < 0.01; ***p-value < 0.001; ns, not significant. [file 13018_2023_4279_MOESM2_ESM.pdf]

A

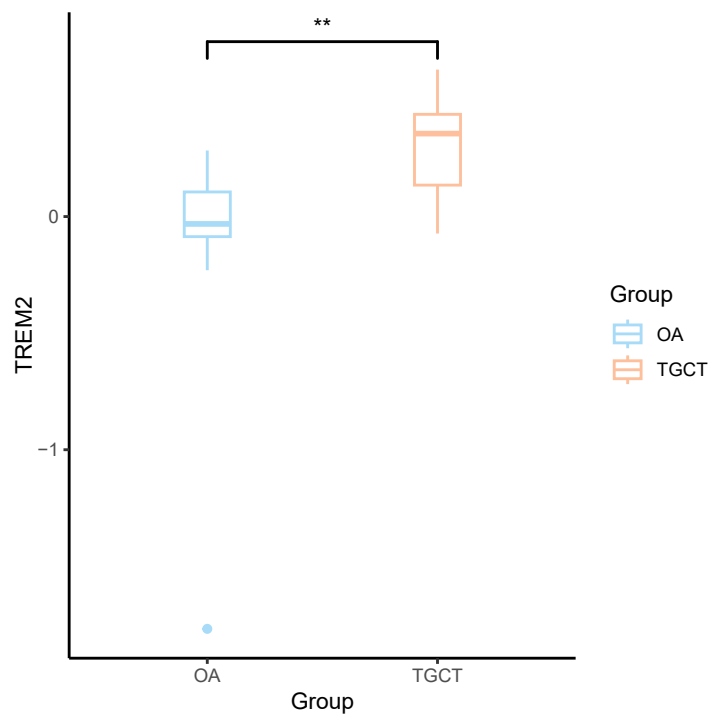

B

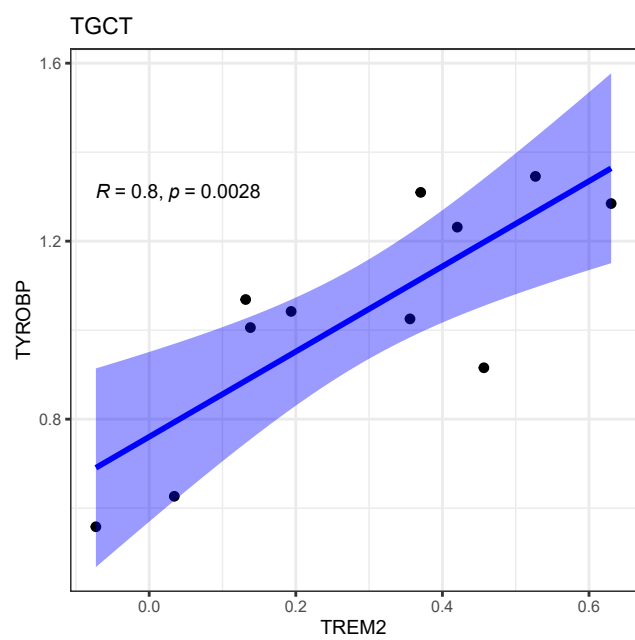

C

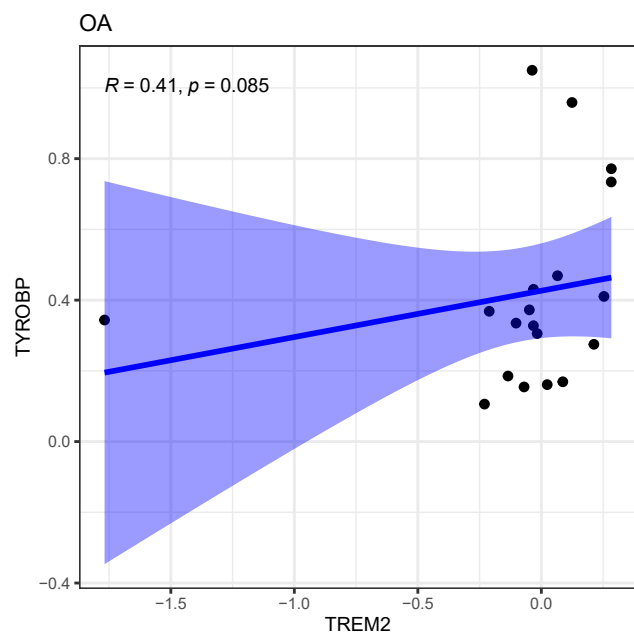

Supplement: Supplementary file 3 — Additional file 3. Fig. S3. (A) The expression level of TREM2 in the GSE3698 dataset. The correlation analysis of the expression of TREM2 and TYROBP in TGCT (B) and OA (C). [file 13018_2023_4279_MOESM3_ESM.pdf]
